# TNFα cleavage beyond TACE/ADAM17: matrix metalloproteinase 13 is a potential therapeutic target in sepsis and colitis

**DOI:** 10.1002/emmm.201302899

**Published:** 2013-06-11

**Authors:** Christoph Becker-Pauly, Stefan Rose-John

**Affiliations:** Biochemical Institute, Christian-Albrechts-University KielGermany

**Keywords:** ADAM protease, inflammatory bowel disease, MMP13, protease web, sepsis

See related article in EMBO Molecular Medicine http://dx.doi.org/10.1002/emmm.201202100

For decades, matrix metalloproteinases (MMPs) have been described as rather unspecific matrix- and collagen-degrading enzymes. Accordingly, this group of proteases was mainly associated with tissue remodeling and pathologic conditions such as tumour development and metastasis. However, broad spectrum MMP inhibitors completely failed in cancer therapy.

Recent studies with gene deficient mice revealed more specific functions of MMPs, *e.g*. the cleavage of chemokines, thereby stimulating inflammatory responses (Dufour & Overall, 2013). Additionally, mass spectrometry-based proteomic techniques allowed for the identification of hundreds of new substrates of MMPs, giving rise to unexpected biological activity of these proteases in health and disease.

MMP13 is known to be one of few collagenases capable of cleaving rigid collagen fibrils. This ability is based on a complex unfolding mechanism of the triple-helical collagen structure. Therefore, MMP13 biology is well studied in bone and cartilage turnover but little is known about other functions.

In this issue, Libert and coworkers report on a pro-inflammatory activity of MMP13 not by ‘simply’ degrading extracellular matrix but through the release of biologically active soluble TNFα from its membrane bound precursor form. Ectodomain shedding of TNFα is a paracrine signaling event in inflammation performed by the metalloprotease tumour necrosis factor α converting enzyme (TACE, also known as ADAM17) (Scheller et al, 2011). Although TACE/ADAM17 is believed to be the major sheddase of TNFα, Vandenbroucke et al demonstrated that after LPS-induced sepsis and DSS-induced colitis, MMP13 is up-regulated and cleaves TNFα. This leads to ER-stress and mucus depletion resulting in an increased interaction of bacteria with the intestinal epithelium. Moreover, elevated levels of bioactive TNFα affect the intestinal permeability through endocytosis of tight junction proteins, thereby increasing systemic inflammation. All these events were abolished in MMP13 deficient mice, resulting in increased survival of MMP13 knockout animals, when treated with LPS or DSS. The authors conclude that MMP13 is a potential therapeutic target for the treatment of colitis (Vandenbroucke et al, 2013).

» …pro-inflammatory activity of MMP13 not by ‘simply’ degrading extracellular matrix but through the release of biologically active soluble TNFα from its membrane bound precursor form.«

TACE/ADAM17 has been identified by its ability to cleave TNFα but later on it turned out that many more proteins are processed by this enzyme. TACE/ADAM17 knockout mice are not viable and show a phenotype strikingly similar to mice lacking ligands of the EGF receptor. All these ligands are transmembrane proteins and need to be shed from the cell surface in order to be systemically active. More than 74 TACE/ADAM17 substrates have been identified (Scheller et al, 2011). Up to now, TACE/ADAM17 was considered to be the only biologically relevant TNFα cleaving enzyme *in vivo* although it was clear that also other proteases such as proteinase-3 could generate biologically active TNFα (Robache-Gallea et al, 1995). Mice, in which the TACE/ADAM17 gene was inactivated only in neutrophils and monocytes/macrophages, failed to shed TNFα upon LPS challenge and were largely resistant against LPS mediated endotoxin shock (Horiuchi et al, 2007).

Since TNFα is involved in the induction and maintenance of many inflammatory diseases and blockade of TNFα is an effective treatment for these conditions, TACE/ADAM17 has been considered a therapeutic target. However, due to the severe phenotypes of mice lacking TACE/ADAM17, treatment of patients seemed to be critical with regard to side effects. Indeed, knock-in mice with 95% decreased levels of TACE/ADAM17 showed increased susceptibility to inflammatory bowel disease, which again is critical for medical treatment (Chalaris et al, 2010). Unexpectedly, Blaydon et al identified a single patient with no functional TACE/ADAM17 due to a mutation in the *Adam17* gene demonstrating that loss of TACE/ADAM17 activity can be compensated in humans (Blaydon et al, 2011).

Libert and coworkers additionally found MMP7 to be up-regulated in DSS-induced colitis in MMP13 knockout mice (Vandenbroucke et al, 2013). MMP7 had previously been shown to induce TNFα-release in a model of herniated disc resorption. However, it is not clear whether this is a direct proteolytic effect on TNFα or if it is mediated through MMP7-mediated stimulation of other proteases. Along the same line, expression of ADAM19, which was also identified as a TNFα-releasing enzyme, is significantly increased in the mucosa of patients with colitis. These studies provide evidence for other proteolytic enzymes that might contribute to TNFα cleavage and progression of colitis and that are possible candidates to compensate lacking ADAM17 activity.

The crucial point in understanding protease networks in terms of compensation is, why MMP13 does not cleave TNFα in TACE/ADAM17 negative mice and why TACE/ADAM17 does not do so in MMP13 negative mice. The most obvious reason is an activation of MMP13 by ADAM17 and vice versa, which is rather unlikely due to their activation mechanisms and the different phenotypes of knockout mice. However, both enzymes might influence the activity of endogenous activators and/or inhibitors, *e.g*. TIMP's, resulting in altered shedding of TNFα when MMP13 or TACE/ADAM17 is deleted. Therefore, it is important to study MMP13 and TACE/ADAM17 expression and activity *in vivo* in corresponding knockout animals with regard to substrate cleavage. This can be challenging, but it is known that protease activity does not necessarily correlate with mRNA or protein levels. For *e.g*. ADAM17 mRNA is expressed virtually in all cells and is not subject to major transcriptional regulation. The protease is, however, mostly located within the cell but is transported to the cell surface mainly in inflamed tissues and in cancer (Scheller et al, 2011).

**Figure 1:**
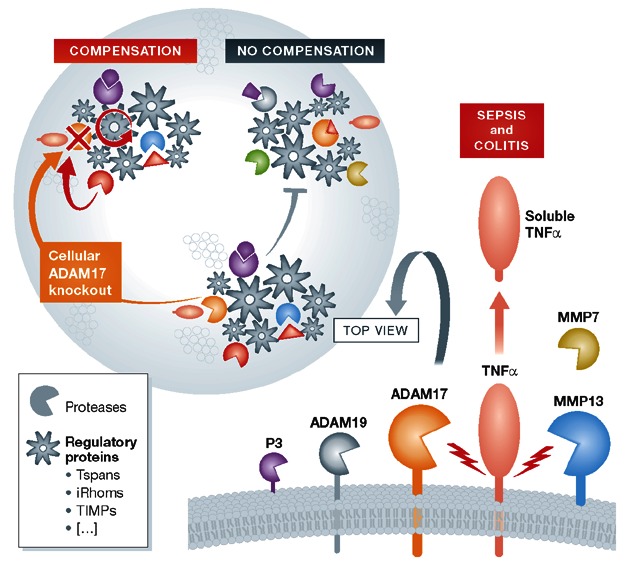
TNFα shedding by MMP13 and TACE/ADAM17 in the protease web.Enhanced ectodomain shedding of TNFα leads to decreased epithelial barrier function, which promotes sepsis and colitis. Different proteases have been identified, however it is still ambiguous which factors under which conditions guide these enzymes to their substrates. Several regulatory proteins, such as tetraspanins (Tspans), inactive rhomboids (iRhoms), or tissue inhibitors of metalloproteinases (TIMPs), might be involved in protease-substrate-interactions. This physically linked protease web has - sometimes but not always — the ability to recognize the loss of single factors, *e.g*. in knock-out cells, which might lead to molecular rearrangements capable of compensating the lack of proteolytic activity. ADAM (a disintegrin and metalloprotease); MMP (matrix metalloproteinase); P3 (proteinase-3).

Are proteolytic enzymes lone fighters or do they have guidance to their substrates in a molecular network that builds the protease web? Could it be that groups of proteases are physically connected in ‘clusters’, either directly or through linker molecules and that these networks rule substrate cleavage? Such a molecular microenvironment has the capacity to register the loss of single players and might then compensate the lack of activity (Fig 1).

An example is the partial compensation of TACE/ADAM17 activity by ADAM10. Blobel and coworkers demonstrated that in embryonic fibroblasts, deletion of TACE/ADAM17 recruits ADAM10 as compensatory sheddase for many substrates (Le Gall et al, 2009). This could be due to the accumulation of uncleaved substrates, which are then processed by other proteases. However, at least in short term treatment, pharmacological inhibition of TACE/ADAM17 in cells also expressing ADAM10 led to no compensation. Consequently, the physical presence of TACE/ADAM17 within the protease web signalizes no change in cell homeostasis, while the lack of TACE/ADAM17 is recognized to stimulate/recruit compensatory proteases. Nevertheless, Blobel and coworkers provided evidence that long-term inhibition of TACE/ADAM17 induced compensation by ADAM10. At this point, however, one should keep in mind that mice lacking ADAM10 or TACE/ADAM17 are not viable demonstrating that compensation is somewhat limited.

A protease network does not explain why MMP13 does not compensate for the loss of TACE/ADAM17 and vice versa. Proteases might be organized in different sub-clusters, meaning that MMP13 and TACE/ADAM17 are not directly linked in the protease web. To clarify this issue, identification of regulatory molecules that orchestrate such protease networks is important. The tetraspanins and iRhoms, the latter being catalytically inactive rhomboid proteases, might be key players in this scenario. It was shown that these transmembrane proteins influence localization and biologic activity of ADAMs and it is likely that other proteases are involved as well (Adrain et al, 2012; Yanez-Mo et al, 2011). Additionally, for numerous secreted proteases tethering to the extracellular matrix was demonstrated, which further supports the hypothesis of clusters of proteolytic enzymes.

Interestingly, only few full knockouts of proteases in animal models lead to severe phenotypes or even lethality. For example, all MMP knockout mice, except of MMP14, reveal fairly mild phenotypic abnormalities, pointing to compensatory mechanisms on mRNA- and/or protein levels, implying activators, endogenous inhibitors, enhancers and other regulatory molecules. This makes it challenging in these animal models to distinguish between molecular differences that are specifically due to the loss of the deleted protease or based on the regulation of compensatory enzymes.

Proteolytic systems are obviously flexible in terms of substrate-cleavage-compensation as demonstrated by Libert and coworkers for the ectodomain shedding of TNFα (Vandenbroucke et al, 2013). Next to the shedding of TNFα by TACE/ADAM17 and MMP13, there are other proteins fitting into this scenario. The amyloid precursor protein (APP) for instance fulfills distinct functions when cleaved by different proteases, at worst leading to neurodegeneration and the development of Alzheimer's disease.

In conclusion, analysing proteolytic events in health and disease with regard to localization, contributing activators, inhibitors, or other regulatory molecules, in one word, the protease web, is important. Anyhow, it will help to elucidate therapeutic strategies under certain pathological conditions and might even help to develop personalized medical treatment.

» …analysing proteolytic events in health and disease with regard to localization, contributing activators, inhibitors, or other regulatory molecules, in one word, the protease web, is important.«
